# Distorted optical input affects human perception

**DOI:** 10.1038/s41598-020-68382-8

**Published:** 2020-07-13

**Authors:** Gad Serero, Maria Lev, Uri Polat

**Affiliations:** School of Optometry and Vision Science, Mina and Everard Goodman, Faculty of Life Sciences, Bar-Ilan University, Ramat Gan, Israel

**Keywords:** Pattern vision, Sensory processing

## Abstract

Collinear facilitation, the mechanism for grouping contour elements, is a process involving lateral interactions that improve the detectability of a target by the presence of collinear flankers. It was shown that the development of collinear facilitation is experience dependent and that it may be impaired when the visual input is distorted in one meridian (meridional amblyopia). In oblique astigmatism, the blurring is on the opposite oblique meridian in both eyes, resulting in two conflicting images, which may affect the development of binocular vision. We hypothesized that the collinear facilitation of adults with oblique astigmatism is reminiscent of the abnormal development of the lateral facilitation of meridional amblyopia. We explored the perception of binocular vision and collinear facilitation in cases of both distorted and non-distorted vision. Fully corrected participants that tested for the target contrast detection of Gabor patches and two collinear flankers, presented for 80 ms, were positioned at different orientations (0° (180°), 45°, 90°, and 135°) and for different eyes (monocular, binocular). The results show a significant anisotropy for monocular collinear facilitation between the blured and the clear meridians, being lower in the blurriest meridian than in the clearest meridian, resembling the meridional amblyopia results. Collinear facilitation results in poor binocular summation between the monocular channels. Our results indicate that the perceptual behavior was similar to that of meridional amblyopic subjects having an anisotropy of collinear facilitation between cardinal meridians in oblique astigmatic subjects.

## Introduction

Normal vision, emmetropia, is when parallel light rays, coming from an object located at more than 6 m away, focus on the retina at the focus point. A refractive error occurs when the light does not focus on the retina due to the shape of the eye. The most common type of refractive error is myopia (nearsightedness), resulting in the perception of far objects as blured because the focus point is before the retina. Astigmatism is a refractive error due to a deviation from the spherical curvature of the cornea and a crystalline lens^1^, resulting in a blured image along the distorted meridian. It occurs when rays propagating in perpendicular planes through the eye are focused at different distances^2^. Thus, the refractive power is different in various meridians and consequently, there is a meridian with a high refractive error and a perpendicular meridian with a weaker refractive error. The distant object has two focal lines perpendicular to the meridians having the maximum and minimum power; thus, two points of focus are formed. Therefore, there are two different images on the retina. Astigmatism can be classified based on its axis orientation. A recent study of Americans showed a prevalence of astigmatism that is 40% of all refractive errors^3^, and another study in China found that the incidence of astigmatism was five time higher in myopic subjects than in non-myopic ones^4^. The most common cases of astigmatism are termed cardinal, which represents 81.6% of the astigmatic population^5^ when the vertical or the horizontal meridians have the highest corneal curvature and thus a stronger refractive power than the meridians do. A less prevalent case is Oblique astigmatism (18.4% of the astigmatic population)^5^, which is characterized by the strongest refractive error having an orientation axis between 16° and 74° or between 106° and 164°. Consequently, the image is blured along the oblique meridians. Note that in most cases of oblique astigmatism the meridians of astigmatism in both eyes show a mirror symmetry^6^, producing two conflicting images, which may pose a problem when combining the monocular inputs. In most cases of cardinal astigmatism, the axes on the retina (blured and clear) are respectively similar and coincide in both eyes, whereas in oblique astigmatism they are not.

**Figure 1:**
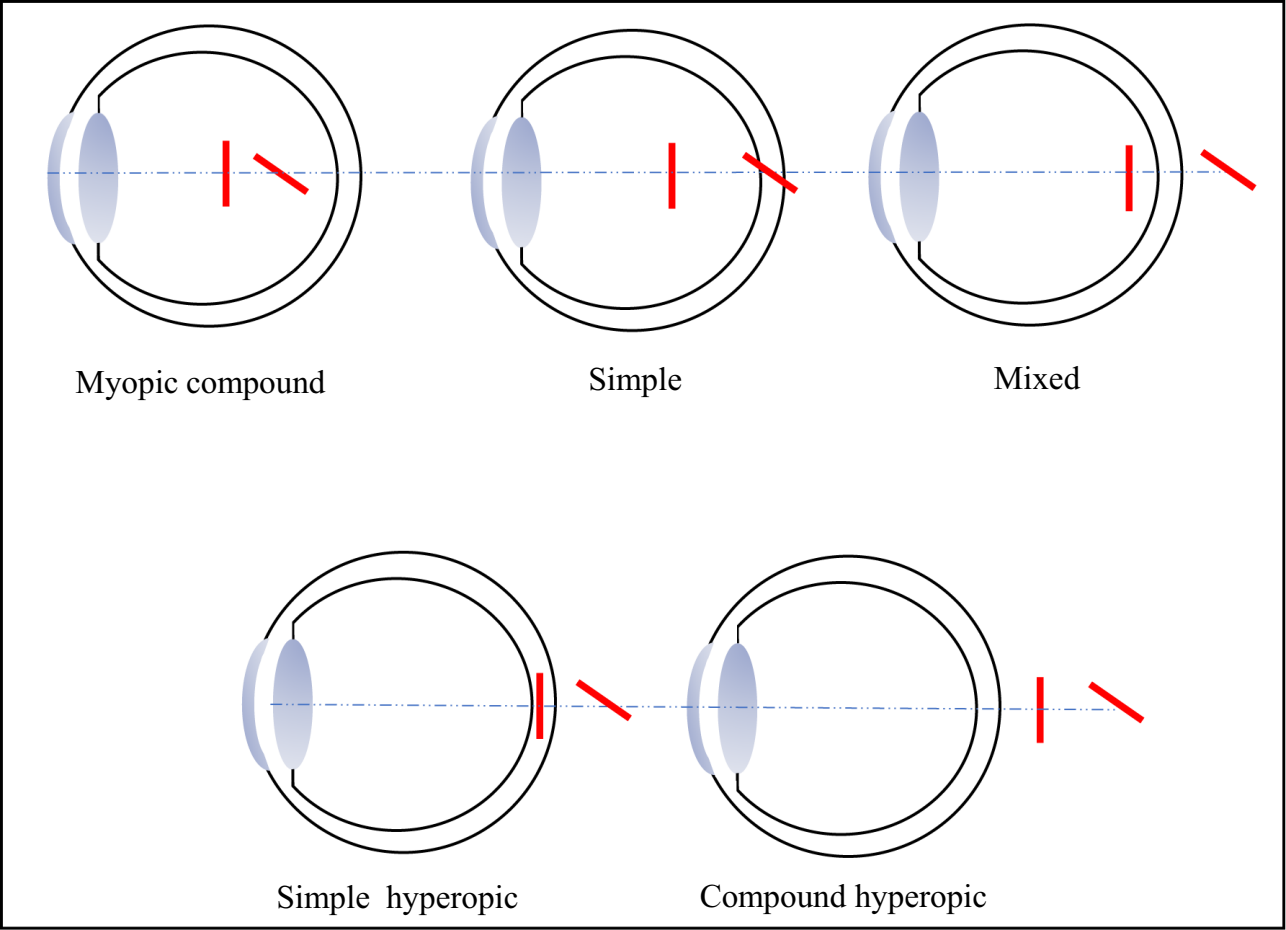
Type of regular astigmatism*.*

Regular ocular astigmatism, when the principal meridian is perpendicular to the second one, has been classified into five sub-categories (see Fig. 1). Myopic compound astigmatism is when the focal points of both focal lines are formed in front of the retina. Myopic simple astigmatism is when one focal line is on the retina and the second is formed in front of the retina. Mixed astigmatism is when one focal line is behind (hyperopic) the retina and one is in front of (myopic) the retina. In hyperopic simple astigmatism the focal line is formed on the retina and the second one is behind it. The last type of regular astigmatism is hyperopic compound astigmatism; it is when both focal lines are formed behind the retina.

Lateral interaction is the ability of a neuron to affect its neighboring neurons by inhibiting or exciting their activity. It was suggested that lateral interactions are shaped and developed during the critical period of development^7^. A specific case of lateral interaction, known as collinear facilitation, is characterized by an improved detectability of a Gabor patch by the presence of collinear flankers^8–10^. The facilitation of target detection increases when flankers are separated from the target by three wavelengths (ƛ) and it decreases for longer distances. Thresholds are elevated (suppressed) for shorter target-to-flanker separations. The choice of this target-flanker distance is determined to create a maximal masking effect on the target^8,11–15^. The reasons for using 3λ separation support the hypothesis that separations of 3λ or more activate collinear facilitation between different neurons responding to the target and the mask. It was shown that collinear facilitation involves horizontal connections between cells of similar orientation preference within V1^16,17^.

Some studies suggested that collinear facilitation exhibits properties reminiscent of the Gestalt Laws of proximity, similarity, smoothness, and good continuation^9,18^ and suggested that it was the mechanism that provides contour integration^19^. Some studies show that the response to a local element in a contour is modified by lateral, suppressive, or facilitative inputs, like the mechanisms of collinear interactions^20^. Other studies^21^ suggest that the sites of the mechanisms responsible for collinear facilitation and contour integration are different.

Normal binocular vision requires two normal monocular visual inputs and the maturation of neural connectivity. Monocular and binocular abnormalities during a sensitive period of development lead to deficient binocular vision^22–25^. The most common abnormal visual inputs leading to amblyopia are strabismus, due to abnormal alignment of the eyes, anisotropy, due to large refractive differences between the eyes, as well as congenital cataracts.

The main phenomenon in binocular vision is binocular summation, which is defined as the superiority of visual function for binocular over monocular viewing^26^. There is wide agreement that luminance and contrast-detection thresholds are approximately 40–60% lower (better) with binocular viewing than with monocular viewing^26–37^. In the last decades, several models of binocular summation have been elaborated^38–40^. The gain control theory model^41^ suggests that each eye exerts gain control on the other eye’s signal in proportion to the contrast energy of its own input and additionally exerts gain control on the other eye’s gain control.

Amblyopia is a developmental disorder associated with early abnormal visual experience that disrupts neural circuitry in the visual cortex; this results in abnormal spatial vision^42,43^ that affects about 3% of the population^44^. It is characterized by reduced visual acuity^42^, binocular dysfunction^45^, reduced contrast sensitivity^46,47^, stereoacuity^48^, phase sensitivity^49,50^, motion perception^51^, shape perception^52^, contour integration^19,53^, visual counting^54^, and spatial interactions^47,55–57^. There are three main types of amblyopia. Anisometropic amblyopia is caused by a robust difference between the refractive errors of both eyes. The second type, strabismic amblyopia, is induced by a strabismus causing a misalignment of the eyes, followed by abnormal binocular stimulation. Deprivation amblyopia is an obscuration of the object by various pathologies such as congenital cataracts^58^. There is a fourth, less common type of amblyopia that occurs in astigmatic subjects. An uncorrected astigmatic refractive error of more than 1.5 diopters during childhood typically results in the development of meridional amblyopia^59^. It occurs because of asymmetrical visual exposure resulting from the astigmatism^59^. Even after optical correction, contrast sensitivity in adults with meridional amblyopia is decreased along the higher optical axis^59^.

During the early period of visual development^60^, asymmetric visual inputs are common but tend to disappear later. Asymmetric visual inputs between the eyes, during the developmental period, are usually caused by a strabismus or a refractive anisotropy, which, without treatment, can lead to amblyopia.

Polat and colleagues^47,61^ examined what happens when collinear facilitation differs abnormally at the axis of higher optical error (cylindrical). They analyzed collinear facilitation as a function of the higher and the lower optical blur axes in humans. Collinear facilitations along the meridian with the lower refractive error (the smallest optical blur) and the meridian with the higher cylindrical error (the higher optical blur) were measured and compared. Interestingly, they found that spatial interactions might be normal in the direction of the lower refractive error, whereas facilitation is poor along the orthogonal axis, where the refractive error is highest. Thus, the process of adjusting to persistently distorted input during development may have a long-term abnormal effect on neuronal connectivity.

A recent study induced cortical distortion in adults using astigmatic lenses to explore the effect of visual distortion on grouping^62^. The results showed that initially there was distortion in the perceived grouping. However, the subjects adapted to the distortion after long experience, and the adaptation was transferred to a long-term memory that can be engaged when blurring is re-applied or disengaged when blurring is removed. This led us to investigate whether astigmatism during the developmental period produces long-term traces that affect the visual perception in adults and to investigate whether the visual perception of astigmatic subjects, especially those with oblique astigmatism, is reminiscent of individuals with meridional amblyopia and whether it may affect their binocular vision.

A second aim of the research was to study the impact of spatial frequency on binocular summation and collinear facilitation. We hypothesized that in subjects with oblique astigmatism the spatial interaction might be different between the two meridians within the same eye. We found that in oblique astigmatic subjects the collinear facilitation was reduced in the blured meridian of astigmatism rather than in the clear one.

## Results

A detailed refractive information is provided in the Methods and Table 1.

**Table 1:** Refractive information.

Subject	Right eye	Left eye
1	pl/− 2.25 × 30	pl/− 2.25 × 140
2	− 1,75/− 1.50 × 70	− 1.25/− 1.00 × 110
3	− 4.25/− 1.00 × 15	− 2.75 × − 2.00 × 150
4	− 4.75/− 0.75 × 130	− 4.00/− 1.75 × 25
5	− 5.00/− 1.25 × 150	− 4.75/− 0.75 × 60
6	− 1.25/− 0.75 × 145	− 1.25/− 1.00 × 30
7	− 0.25/− 1.00 × 120	pl/− 0.75 × 40
8	− 1.50	− 1.25/− 0.75 × 60
9	− 2.00	− 0.50
10	− 1.00	− 1.00
11	pl	pl
12	pl	− 0.25
13	− 4.00	− 4.25
14	− 3.25	− 3.25
15	− 0.50	− 0.75
16	− 0.75	− 0.75
17	− 1.75	− 1.25
18	− 2.50/− 0.50 × 100	− 2.75/0.75 × 105
19	− 4,75/− 0.50 × 105	− 4.25/− 1.00 × 85
20	− 3.00/− 1.00 × 180	− 3.00/− 1.00 × 180
21	− 1.00/− 1.25 × 95	− 0.75/− 1.00 × 90

The refractive information for each subject was measured before the experiment. The experiment was performed using full optical correction.

### Single target

#### Single target threshold

**Figure 2:**
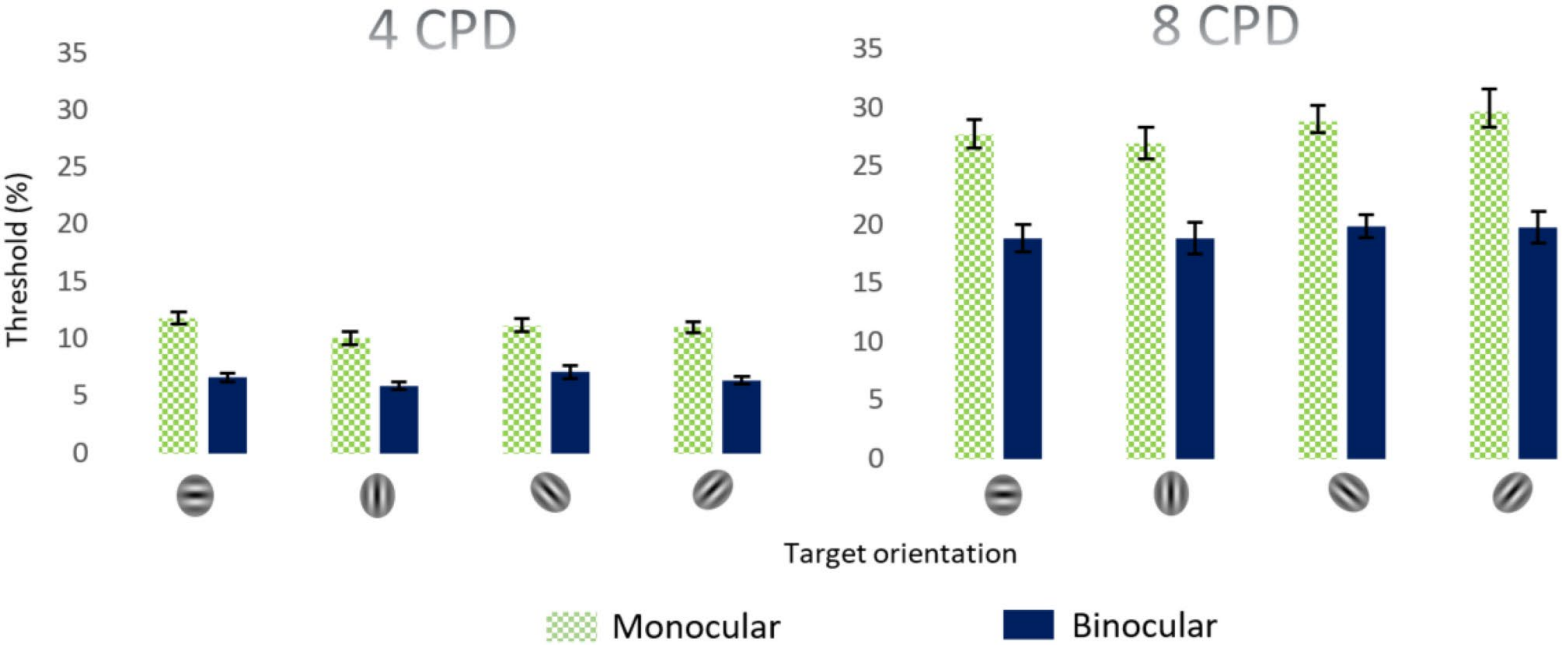
Monocular and binocular single-target thresholds according to orientation and spatial frequency**.** The orientations are respectively represented on the abscise by 180°, 90°, 135°, and 45° at 4 and 8 cpd. Error bars represent the standard error of the mean. (n = 21).

First, we measured single target contrast thresholds according to orientation. Figure 2 presents the contrast threshold of a Gabor target, under monocular and binocular conditions, presented at four orientations (180°, 45°, 135°, and 90°) for a spatial frequency of 4 cpd. The difference between the target’s contrast thresholds of the three groups, for all orientations, was not significant (*p* = 0.18, 2-way ANOVA), indicating no anisotropy of the target’s threshold as a function of orientation. Thus, we decided to collapse the results of the 3 different groups of the study (21 subjects), 9 with oblique astigmatism, 4 with cardinal astigmatism, and 8 with spherical correction (no astigmatism). The results for monocular conditions are denoted in green and those for binocular conditions are in blue.

#### Binocular summation of a single target

We calculated the binocular summation as the ratio between the contrast thresholds of the average of two monocular eyes to the binocular threshold.[1-(Binocular Threshold/ Monocular average threshold)) × 100; (%)]^26^. As expected from previous literature^26,49^, we found a significant difference between the monocular and the binocular single target contrast threshold (42% ± 3.8; *p* = 0.0003; mean ± SE; paired 2-tailed t-test, post hoc test, 2-way ANOVA), which could be explained by the binocular summation effect. Interestingly, our results did not show the effect of orientation on binocular summation (*p* = 0.1, 2-way ANOVA). The binocular summation of the 3 study groups was similar (mean ± SE; 41.5% ± 5.7); then we decided to collapse the results.

#### High spatial frequency

At 8 cpd, similar to 4 cpd, the monocular and binocular single-target thresholds for all orientations were not significantly different for both conditions (*p* = 0.46, *p* = 0.53, 2-way ANOVA). However, as expected, we found a robust increase in single-target thresholds, compared to at 4 cpd (mean ± SE; Monocular: 28.7% ± 3.4, *p* = 0.004; Binocular: 18.93% ± 3.8, *p* = 0.005). Interestingly, this increase was not affected by orientations. Contrary to our previous expectations^63^, based on the quadratic summation prediction^28^, we found a significant decrease in binocular summation (*p* = 0.04, paired 2-tailed t-test) by 10% at the higher spatial frequency.

### Collinear facilitation and astigmatism

#### Collinear facilitation according to the meridians of astigmatism

To explore the impact of astigmatic refractive error on collinear facilitation, we measured collinear facilitation according to orientation. After matching the stimuli’s orientation with the meridian of astigmatism of each eye, we calculated the collinear facilitation for the orientation stimuli matched either with the blured or with the clear corneal astigmatic meridian.

**Figure 3:**
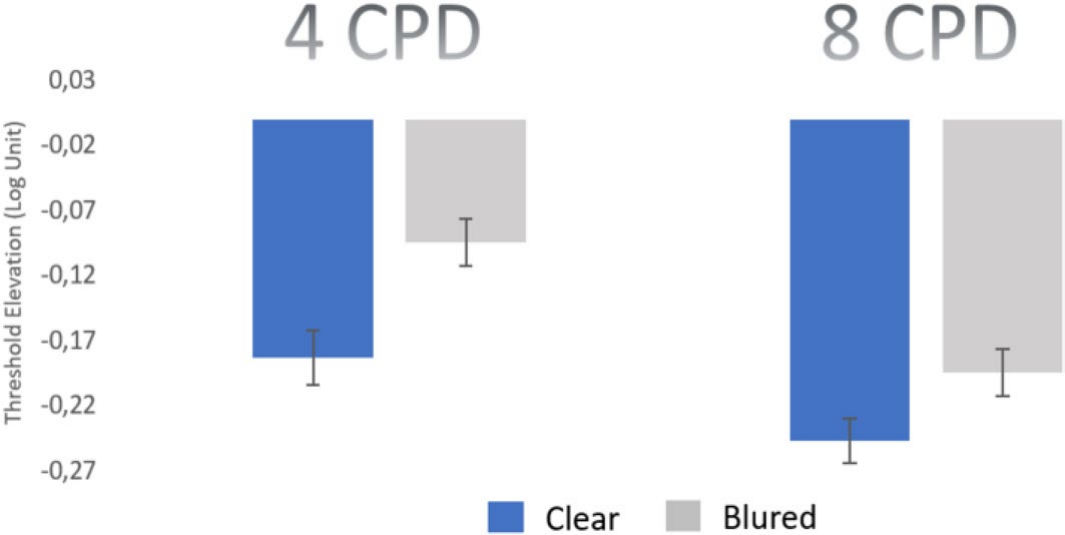
Monocular collinear facilitation for oblique astigmatic subjects. At 4 cpd there is a significant collinear facilitation and anisotropy between the clear and the blured meridian. When the orientation of the target’s stimulus matches the stimulus orientation, facilitation is significantly higher than with the blured meridian (*p* = 0.003, paired 2-tailed t-test). At 8 cpd, the anisotropy is significantly maintained but it is lower (*p* = 0.04, paired 2-tailed t-test). At 8 cpd, there is a significant increase in facilitation for both meridians (*p* = 0.018, *p* = 0.016, paired 2-tailed t-test). Error bars represent the standard error of the mean. n = 8.

In cardinal astigmatic subjects, we did not find a significant anisotropy in the collinear facilitation between the blured and the clear meridians (*p* = 0.535, paired 2-tailed t-test). However, we did find a significant anisotropy in the collinear facilitation (see Fig. 3) in oblique astigmatic subjects, between the blured and the clear meridians. When the global orientation of the stimuli matched the blurriest meridian, facilitation was lower than when it matched the clearest meridian (mean ± SE; blur − 0.09 ± 0.02 log units; clear, − 0.018 ± 0.02 log units, *p* = 0.003, paired 2-tailed t-test). This result is consistent with a previous study in amblyopia showing that facilitation is reduced along the blured meridian when it matches the highest refractive error^47^. At 8 cpd, this anisotropy is significantly maintained but it is lower (*p* = 0.04, paired 2-tailed t-test).

#### Binocular summation of collinear facilitation

**Figure 4:**
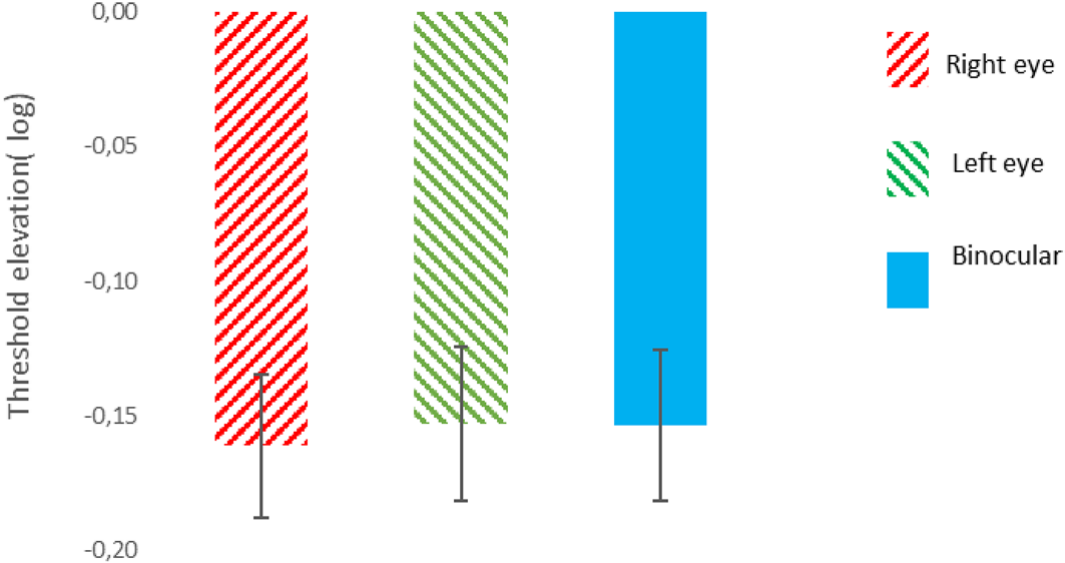
Binocular summation of collinear facilitation. The error bar represents the standard error of the mean. (n = 21).

We compared monocular to binocular collinear facilitation on oblique targets. Based on predictions of the binocular summation models^63^, since the contrast thresholds under binocular facilitation should be lower, threshold under binocular facilitation should be lower (more facilitation) than the average thresholds under monocular facilitation. However, our results show that there is no advantage in binocular facilitation, and they indicate that binocular facilitation is approximately equal to the amount of facilitation of the eye with the better collinear facilitation, i.e., for the orientation’s stimuli that match the clearer astigmatic meridian (see, Fig. 4) (mean ± SE; Right eye = − 0.16 ± 0.02 log units, Left eye = − 0.15 ± 0.02 log units; Monocular = − 0.155 ± 0.03 log units; Binocular − 0.15 ± 0.03 log units), *p* = 0.18, *p* = 0.54, paired 2-tailed t-test). In other words, our data indicate that there is no sign of binocular summation of collinear facilitation, reminiscent of the results found for amblyopia^61^. We suggest that binocular summation of collinear facilitation is not linear and that it is consistent with the concept of the cyclopean image theory of binocular combination, suggesting that for stimuli of ordinary contrast, when either eye is stimulated alone, the predicted binocular contrast is the same as when both eyes are stimulated equally^41^.

### Masked targets

#### Collinear facilitation

We next tested the effect of collinear facilitation on the 4 different orientations under monocular and binocular conditions. Briefly, we measured collinear facilitation separately for the 3 study groups, calculated as the log ratio of the thresholds of single targets and masked targets. Collinear facilitation was similar between the spherical and cardinal astigmatic groups (but not for the oblique group, see below), with no significant difference between orientations and threshold (monocular *p* = 0.84, binocular *p* = 0.55). Therefore, we decided to collapse the results of the collinear facilitation of the sphere and the cardinal and to consider them as a control group.

#### Effects of orientation on collinear facilitation

**Figure 5:**
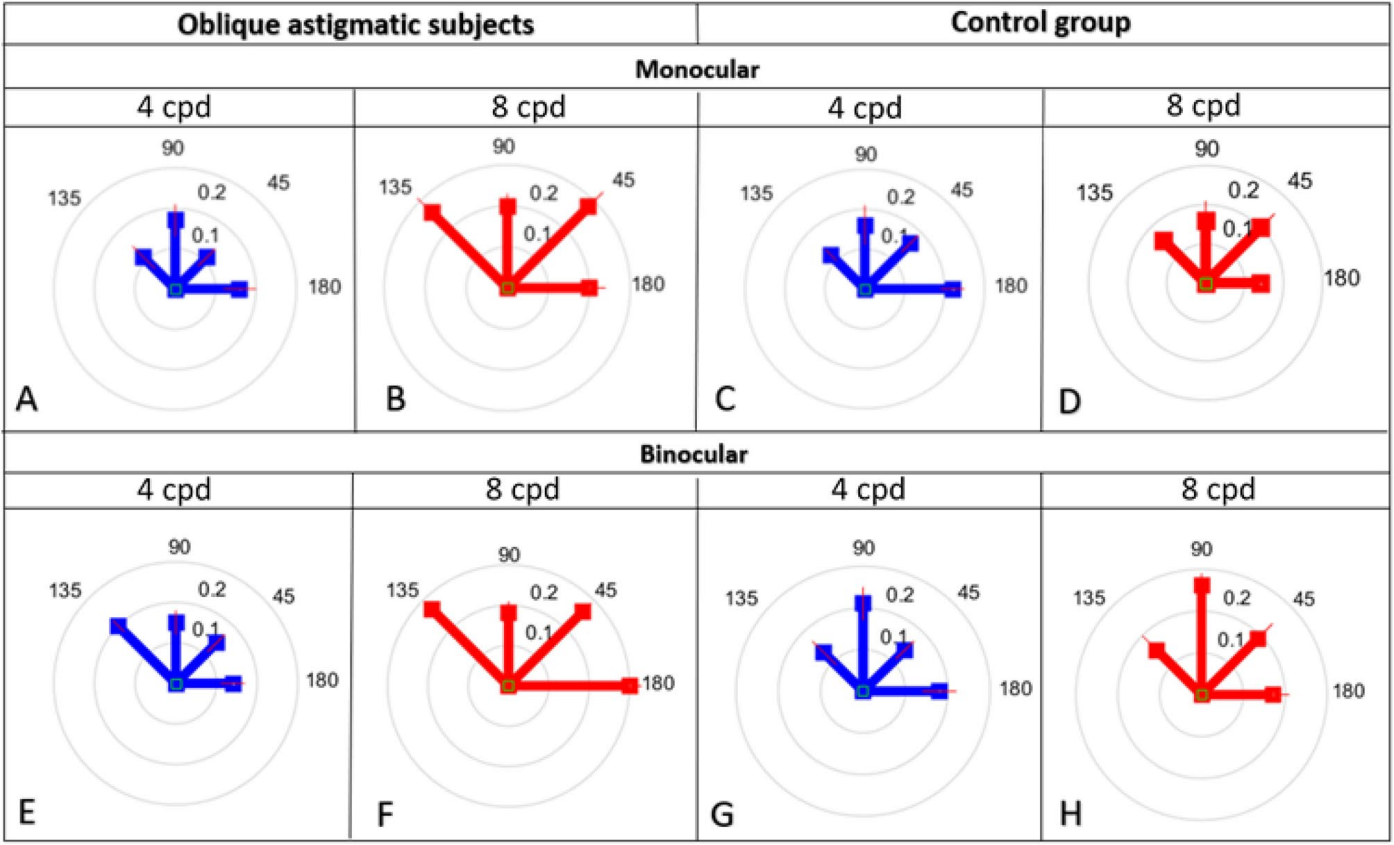
Collinear facilitation (CF) according to orientation. **a** Monocular CF for oblique astigmatic subjects at 4 cpd. **b** Monocular CF for oblique astigmatic subjects at 8 cpd. **c** Monocular CF for the control group at 4 cpd. **d** Monocular CF for the control group at 8 cpd. **e** Binocular CF for oblique astigmatic subjects at 4 cpd. **f** Binocular CF for oblique astigmatic subjects at 8 cpd. **g** Binocular CF for the control group at 4 cpd. **h** Binocular CF for the control group at 8 cpd. Each circle indicates the CF in log units (0.1, 0.2, and 0.3).

In Fig. 5 we collapsed the data for the astigmatism axis for ± 15 degrees from the subject’s axis in order to remain with only 4 main orientations. Figure 5a shows a significant monocular collinear facilitation and anisotropy between the cardinal stimulus orientation (180°and 90°) and oblique (135° and 45°) in oblique astigmatic subjects at 4 cpd. In fact, we found that collinear facilitation was higher in the cardinal than in the oblique stimulus orientation. At 8 cpd, we observed a robust general increase in collinear facilitation with no difference between orientations. However, this effect was not consistent for control group subjects. Under other conditions we did not observe a major and significant effect of orientation between the monocular and binocular conditions even at higher spatial frequencies.

#### Monocular condition

Our results show (Fig. 5a) a significant anisotropy of collinear facilitation between the cardinal and oblique orientation at 4 cpd (mean ± SE; cardinal: 0.165 ± 0.04 log units; oblique: 0.11 ± 0.04 log units; *p* = 0.05; paired 2-tailed t-test). In fact, collinear facilitation is reduced (*p* = 0.04) by 0.1 log units for oblique compared with cardinal meridians at 4 cpd (see Fig. 5a). However, at 8 cpd (Fig. 5b), collinear facilitation increased significantly (*p* = 0.05) at 4 orientations. As shown in Fig. 5c and d, there was no significant difference in monocular collinear facilitation for the control group after increasing spatial frequency, for all orientations (*p* = 0.68), suggesting the existence of an oblique effect for collinear facilitation at 4 cpd. This oblique effect was observed in the control group between the cardinal and oblique orientations, but it was not statistically significant (mean ± SE; cardinal; 0.16 ± 0.03 log units; oblique: 0.13 ± 0.04 log units; *p* = 0.08; paired 2-tailed t-test). Figure 5b shows that at 8 cpd there was a significant increase in collinear facilitation only for the oblique orientation for the oblique astigmatic group, compared with the control group (Fig. 5d) (mean ± SE ;cardinal: 0.165 ± 0.04 log units; oblique: 0.11 ± 0.04 log unit; *p* = 0.05; paired 2-tailed t-test).

### Binocular condition

Figures 5e and f show that binocular collinear facilitation increases significantly by (mean ± SE) 0.1 ± 0.01 log units (*p* = 0.04, paired 2-tailed t-test) with no effect of orientation (*p* = 0.4; 2-way ANOVA) for higher spatial frequency in all orientations for oblique astigmatic subjects. Interestingly, this effect did not occur in the control group (*p* = 0.48) including orientation (*p* = 0.52). However, we noticed the absence of an oblique effect for the binocular condition at 8 cpd in the oblique astigmatic but not in the control group (Fig. 5g and h). The low oblique effect observed in our study at 8 cpd could be explained by the small size of the target Gabors used^64,65^.

## Discussion

The aim of our study was to explore the influence of oblique astigmatism on perception, emphasizing binocular summation and collinear facilitation. Whereas the binocular summation for a single target was as expected (about 40%), with no difference between the orientations, no significant binocular summation of collinear facilitation was observed. When the orientation’s stimuli matched the orientation of the clear meridian, the obtained monocular collinear facilitation was significantly higher than when the orientation’s stimuli matched the blurriest meridian orientation (*p* = 0.02, paired-2-tailed t-test). Interestingly, a summation of binocular facilitation does not follow the expected summation of the two monocular facilitatory thresholds; however, it was the same as the monocular threshold of the eye whose orientation’s stimuli matched the clearest meridian. For example, if the orientation’s stimuli and the clearest meridian of the right eye are both oriented at 135°, the blurriest meridian of the left eye will be 135°. Indeed, according to our results, the binocular and monocular collinear facilitation thresholds, represented by the ratio (flank thresholds/target threshold in log units), were equal, showing a quasi-inexistent binocular summation.

### Pseudo-amblyopic behavior

Polat et al.^47^ showed that lateral interaction in meridional amblyopic subjects decreased when stimuli were presented in the orientation at the blurriest meridian in the amblyopic eye. In our study, in oblique astigmatic subjects, with normal visual acuity, when stimuli were oriented in the same orientation as the blurriest meridian, we observed a decrease in the facilitation by 13%, compared with the clear meridian (*p* = 0.04, paired t-test). These results were not found in the other group of cardinal astigmatic subjects. This effect led us to suggest that a similar mechanism of poor collinear facilitation exists for meridional amblyopia and oblique astigmatism. It is important to recall that this result is obtained despite that all our subjects (oblique, cardinal astigmatic, and spherical) have fully refractive correction with vision of 20/20 or better and with no signs of amblyopia or refractive anisotropy.

### Grouping and adaptation to blurring

Previous studies^62^ showed that adaptation to astigmatic blur is transferred to long-term memory that could be engaged when blur is re-applied or disengaged when blur is removed. In our study, we asked the subjects to specify whether they wear glasses during their sensitive period; most of them were unable to provide accurate information. However, when they used their best optical correction (reaching visual acuity of 6/6 or better), which is supposed to eliminate the blurring, there are still perceptual monocular markers regarding the level of monocular collinear facilitation.

### Binocular summation and spatial frequency

One purpose of our study was to examine the impact of changing spatial frequency on binocular summation. Studies have shown that contrast sensitivity is reduced with increasing spatial frequency^66^, especially in amblyopia^67^. Baker et al.^63^ have shown that binocular summation is significantly affected by the spatial and temporal frequency of the stimulus. They defined a measure of “stimulus speed” that they calculated as the ratio between stimulus temporal (presentation time) and the spatial frequency. According to this ratio, a slow speed (including high spatial and low temporal frequencies) will lead to a higher binocular summation. For all the groups, our results showed a general decrease in binocular summation for the lower stimulus speed (i.e., a higher cpd). However, these results contradict the expectations based on the suggestion that high spatial frequency increases binocular summation^63^. This difference could be explained by the different spatiotemporal properties of stimuli used in our experiment. It is important to note that the stimuli were presented for a rectangular 80 ms pulse. This would make them broadband in temporal frequency. Here, the comparison of stimulus speed may be less relevant for our study. We also noted that we used a local Gabor patch as a stimulus, compared to a sine wave grating stimulus with a higher number of cycles, which might not be smoothed by a Gaussian filter, whereas most of the studies used a single E target, with differences in size, times of exposure, and contrast^63^.

### Individual differences in interocular sensitivity

Interocular differences in sensitivity may be an important factor that can affect binocular summation. Studies showed that the high imbalance of sensitivity between the eyes at higher spatial frequencies may influence the level of binocular summation. The model of Ding and Sperling^41^ suggests that each eye exerts a gain control on the other eye in proportion to its total contrast energy. Thus, we suggest that the decrease in binocular summation that we found may result from a binocular imbalance, due to the low inhibition exerted by the “weaker’’ monocular channel on the other one. In our study we found a monocular threshold imbalance in three subjects of the control group at 8 cpd, which did not exist at 4 cpd. The binocular threshold was similar to the better monocular threshold, suggesting a poor binocular summation either due to the high inhibition of the strong monocular channel, or due to poor inhibition of the weaker one.

### Binocular collinear facilitation

One of the main purposes of our research was to study the impact of spatial frequency on binocular summation and collinear facilitation. Confirming the previous studies^47^, we found significantly higher monocular and binocular thresholds for a single target at 8 cpd in all groups. However, the higher level of collinear facilitation was found only for the oblique astigmatic group in all meridians. Thus, we suggest that for oblique astigmatic subjects the monocular and binocular channel interacts differently at higher spatial frequencies by reducing inhibition. Given the fact that there is less binocular summation (facilitation), it could be due to additional inhibition, probably due to inter-ocular suppression that acts to cancel the binocular facilitation^68, 69^.

Our results show that at 8 cpd there is a significant increase in the collinear facilitation (monocular and binocular). Interestingly, under both conditions, the thresholds of the binocular and monocular collinear facilitation are similar to the monocular facilitation of the eye with the higher amount of collinear facilitation, whose orientation stimuli matched those of the clearest meridian. Thus, these results suggest that there is no real binocular summation, and that the binocular percept is determined mainly by the monocular percept. This effect could be due to an interocular inhibition process between the monocular channels, in the processing of the total collinear facilitation from each ipsilateral channel, in agreement with the Ding and Sperling^41^ model of binocular summation.

Huang et al.^21^ studied the effect of monocular vs binocular facilitation. The binocular conditions were either purely binocular (the target appears at the same eyes as the flankers) or dichoptic (the target appears at different eyes than the flankers). Models of binocular summation predict more summation with lower contrast. When the target’s contrast under collinear facilitation is lower than that of the isolated target, then one should expect more summation for collinear facilitation. However, our results and the results of Huang et al.^21^ did not show any further facilitation (summation) under the binocular condition. Moreover, the results of Huang et al.^21^, under the dichoptic condition, show no collinear facilitation at all. Thus, the existing data do not support the idea of binocular summation of collinear facilitation.

Note that our study did not test the collinear facilitation at target-flanker separations of more and less than 3λ. It has been shown that the size of the human perceptive field increases with increasing eccentricity and that the effect of masking is related to the size of the perceptive field^15^. It is also suggested that the size of the suppression zone, which may be related to the size of the perceptive field, is larger in amblyopia^15^. It would be interesting to determine whether the anisotropy of collinear facilitation could vary with increasing or decreasing target-flanker separation. In normal vision, using 3λ flanker separation provides more activation of collinear facilitation between different neurons responding to the target and the mask (lateral masking); thus, the collinear facilitation is the most prominent factor for a target-to-flanker separation of 3λ; however, it decreases for longer distances^8^.

## Conclusion

According to previous studies, and to our results with oblique astigmatic subjects, we suggest that early visual experience may induce abnormal anisotropy that is manifested as perceptual behavior in adulthood, which mimics amblyopia, as manifested by reduced collinear facilitation. In a clinical setting, oblique astigmatism patients find it hard to adapt to an oblique cylinder needed for refractive correction. Thus, we suggest that the anisotropy in meridional collinear facilitation observed in our study could represent a “cortical trace” induced by an atypical visual experience in oblique astigmatism during the sensitive period, which is retained in adulthood.

## Methods

### Participants

A total of twenty-one subjects were enrolled in the study. This includes a group of eight subjects with oblique astigmatic error (n = 8) and a control group of thirteen subjects either without astigmatic error (n = 9) or with cardinal astigmatic error (n = 4). In order to minimize the accommodation, all our subjects were emmetropes, myopes, or with simple and compound astigmatism. The age of the subjects was between 18 and 30 years old (27.1 ± 4.98 years, mean ± standard deviation); they signed a consent form that was approved by the Internal Review Board (IRB) of Bar-Ilan University and all methods were performed in accordance with the relevant guidelines and regulations and each subject was included only after 'informed' consent have been obtained. Also each subject was included after passing a full optometric eye exam, fully corrected with no amblyopia or ocular disease and with visual acuity of 6/6 (20/20) or better.

In addition, each subject passed a full optometric exam including visual acuity based on Snellen and logMar charts (ETDRS), autorefraction, retinoscopy, subjective, and binocular tests (Cover test), stereo vision (Random dot), Van Graef, fusional reserve, amplitude of accommodation, as well as negative and positive relative accommodation. As part of the clinical procedure of the optometric exam, the refractive power of each meridian (astigmatism) was determined and used for the experiment.

### Apparatus

The stimuli were presented using a PC computer controlled by a NVDIA GTX 710 video card and a BENQ XL 2,411 color monitor using custom software PSY developed by Yoram S. Bonneh^70^. The screen resolution was 1920 × 1,080 pixels and gamma correction was applied. The effective size of the monitor screen was 52 by 30 cm, which, at a viewing distance of 100 cm, subtended a visual angle of 29° × 17°. We used 3D vision wireless stereoscopic polarized goggles (NVIDIA 3D stereoscopic glasses) that provided direct synchronization with the stimuli; thus, the subjects were unaware of the stimulated eye. The screen background luminance was synchronized with the NVIDIA glasses in order to maintain a background luminance of 40 cd/m^2^. A dark polarized lens was used. However, it led to a higher single target threshold level.

### The stimuli

**Figure 6:**
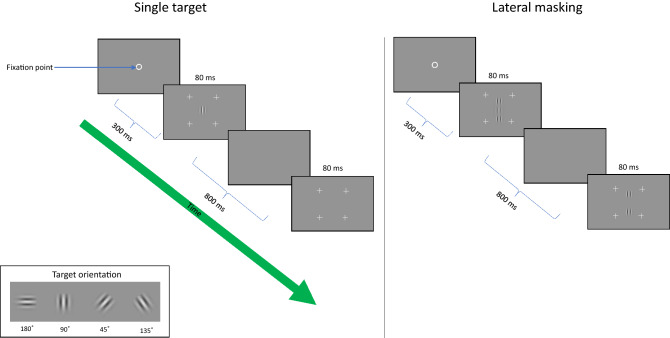
The lateral masking paradigm. Participants are required to decide which interval (the first or the second) is marked by four peripheral crosses. The central Gabor target was presented. The contrast threshold of the target was measured using an adaptive method to reach a 79% correct threshold.

Stimuli were localized gray-level gratings (Gabor patches, Fig. 6) with an equal wavelength (λ) and standard deviation (standard deviation, σ), allowing a minimum of 2 cycles. The Gabor patches were modulated from a background luminance of 40 cd/m^2^ (measured with the stereoscopic glasses). Eight-bit depth was used for rendering the Gabors along with 1% minimum presentation contrast. We used two spatial frequencies: 4 and 8 cycles per degree (cpd, λ = 0.433° and 0.21°, respectively). The stimuli were presented in four orientations: 0° (180°), 45°, 90°, and 135° (see Fig. 6) and for 80 ms. A two-temporal alternative forced-choice paradigm and a 3:1 staircase procedure, known to converge to 79% correct response, were used to measure the target contrast detection threshold^71^. In this method, the target contrast is increased by 0.1 log units (26%) after an erroneous response, and it is decreased by the same amount after three consecutive correct responses. In each block, there are 8 reversal steps; the threshold is determined by the average of the last 6 reversals.

### The lateral masking paradigm

Participants started each trial by pressing the middle mouse button. A visible fixation circle was presented in the center of the screen until the participants pressed the button again to start the intervals. The two intervals were 60 ms each with an 800 ms gap between them. The first interval was preceded by a 300 ms blank period with a temporal jitter of 500 ms, on average. The target GP was presented in only one of the intervals (order randomized). Participants were asked to report which interval contained the target by pressing a mouse button (left for the first interval and right for the second). Across trials, target presentation was equally distributed between the two intervals. Participants were instructed to maintain their fixation in the center of the monitor and to avoid eye movements during the trials.

Four to 5 meetings were needed to collect all the data. The first day was dedicated to a full optometric eye exam. The remaining meetings of two hours each were dedicated to run a total of 48 blocks, depending on the attention capacity of the subject. Each orientation was presented randomly. Each subject was asked to repeat the set of 24 blocks, including a maximum of 80 trials per block. The size of the stimuli for target-flanker separations of 3 λ (center-center) subtends a visual angle of about 1.66 ° in the central visual field. For each orientation, two conditions were tested. One measured the contrast detection threshold of a single foveal target (T) and the other, the contrast detection threshold of T in the presence of two collinear flankers having a contrast of 40% (lateral masking, LM). The T and the masks were separated from each other by 3 wavelengths (λ).
